# Ultrasonographic evaluation of the fetal central nervous system: review of guidelines

**DOI:** 10.1590/0100-3984.2018.0056

**Published:** 2019

**Authors:** Hérbene José Figuinha Milani, Enoch Quindere de Sá Barreto, Edward Araujo Júnior, Alberto Borges Peixoto, Luciano Marcondes Machado Nardozza, Antonio Fernandes Moron

**Affiliations:** 1 Department of Obstetrics, Escola Paulista de Medicina da Universidade Federal de São Paulo (EPM-Unifesp), São Paulo, SP, Brazil.; 2 Department of Obstetrics and Gynecology, Universidade Federal do Triângulo Mineiro (UFTM), Uberaba, MG, Brazil.

**Keywords:** Fetus, Central nervous system, Ultrasonography, Practice guidelines as topic, Feto, Sistema nervoso central, Ultrassonografia, Guias de prática clínica como assunto

## Abstract

Central nervous system malformations constitute the second most common group of anomalies in fetuses. Such malformations have assumed clinical importance because of their association with high rates of perinatal morbidity and mortality. Therefore, it is extremely important to assess the fetal central nervous system during the prenatal period, in order to identify any changes in its development and thereby gain sufficient information to advise parents about pregnancy follow-up, options for fetal therapy, and the timing/type of delivery, as well as the postnatal treatment and prognosis. The objective of this review was to describe the ultrasonographic evaluation of the fetal central nervous system as per the guidelines of the International Society of Ultrasound in Obstetrics and Gynecology.

## Abstract (pt)

Malformações do sistema nervoso central são o segundo mais frequente grupo de anomalias que afetam o feto. Elas têm adquirido grande importância em razão da sua associação com altas taxas de morbidade e mortalidade perinatal. Além do mais, é extremamente importante avaliar o sistema nervoso central fetal durante o período pré-natal, de modo a se diagnosticar possíveis mudanças no desenvolvimento e aconselhar os casais sobre o seguimento da gestação, possibilidades terapêuticas fetais, tempo e tipo de parto, tratamento pós-natal e prognóstico. O objetivo desta revisão foi descrever a avaliação do sistema nervoso central fetal por meio das recomendações da International Society of Ultrasound in Obstetrics and Gynecology.

## INTRODUCTION

Congenital central nervous system (CNS) malformations constitute a common group of anomalies in fetuses, second only to cardiac malformations. The incidence of congenital CNS malformations ranges from 1 to 2 cases per 1000 births^(^^1^^)^, and its epidemiology is influenced by environmental and genetic factors, as evidenced by geographical variations in its incidence^(^^2^^)^. Such malformations have clinical importance because they are associated with high rates of morbidity and mortality, influencing the neurocognitive and motor development of the survivors, who may have lifelong sequelae. Therefore, it is extremely important to assess the fetal CNS during the prenatal period, in order to identify any changes in its development and give appropriate advice to parents regarding pregnancy follow-up, options for fetal therapy, and the timing/type of delivery, as well as the postnatal treatment and prognosis.

The evaluation and diagnosis of CNS malformations during the prenatal period can be performed by ultrasound at any gestational age. The ultrasonographic evaluation includes the study of the brain and spinal cord. It is important to determine whether the CNS structures present complex embryology and anatomy, because the CNS undergoes most of its changes during gestation. All CNS structures are derived from the three primary brain vesicles (the prosencephalon, mesencephalon, and rhombencephalon). At the end of the first trimester, the choroid plexuses occupy almost all of the cerebral hemispheres, which are already separated by the interhemispheric fissure. This stage is also marked by the formation of the cerebral ventricular system and, as gestation progresses, the size of the lateral ventricles and choroid plexuses decrease in proportion to that of the brain. Development of the cerebellum also occurs with the closure of the cerebellar vermis. The corpus callosum shows growth in the anteroposterior direction, with differentiation into its segments (rostrum, genu, body, and splenium). The cerebral cortex undergoes a complex process of development, with the formation of sulci and gyri during gestation. The formation of a complex neural network occurs through the process of proliferation, migration, and organization of neurons. The spinal cord undergoes an ascension process in relation to the spine^(^^3^^)^. All of these changes are associated with changes in the ultrasound aspects of the CNS during gestation. Therefore, every professional involved in fetal evaluation (specialists in fetal medicine, radiologists, and obstetricians) should be aware of the embryology and anatomy of the fetal CNS, as well as of its ultrasound characteristics at different gestational ages, in order to avoid diagnostic errors. In addition, it is of fundamental importance to understand the congenital malformations that can affect the CNS regarding the following aspects: ultrasound manifestations; physiopathology; prognosis; and prenatal/postnatal follow-up.

Some abnormalities can be diagnosed in the first trimester, although such abnormalities represent only a minority of the potential malformations and are usually the most severe (e.g., acrania and alobar holoprosencephaly). Ultrasonographic evaluation of the fetal CNS in the first trimester is usually performed in the axial, sagittal and coronal planes, using abdominal and transvaginal approaches. In the first trimester, it is also possible to identify the following structures: the cranium (ossification of the skull, allowing evaluation of its contour, shape and integrity, occurs at approximately 10 weeks of gestation); the interhemispheric fissure (present from 10 weeks of gestation); the choroid plexus; the thalamus; the posterior fossa (intracranial translucency being an important marker of spina bifida and posterior fossa malformations); and the spine.

Most efforts to diagnose CNS malformations occur during the second trimester, in the examination of fetal morphology conducted at 20-24 weeks of gestation^(^^4^^)^. Given the characteristics of brain development described above, some features are susceptible to changes throughout gestation, mainly secondary to the effect of external agents such as infection, trauma, and hemorrhage. It is therefore important to emphasize that a normal CNS assessment in the second-trimester morphology scan does not exclude the emergence of fetal alterations during pregnancy. Hence, it is necessary to re-evaluate the fetal brain morphology throughout a pregnancy^(^^5^^)^.

Conventionally, the ultrasound evaluation of brain development during pregnancy is performed in the axial planes of the fetal skull. However, that type of evaluation has some limitations. For instance, the attenuation of the sound beam by the skull can impair the evaluation of the cerebral hemisphere proximal to the transducer, and, because the brain is a three-dimensional organ with a complex anatomy, the midline structures, such as the corpus callosum, the brainstem, the cerebellar vermis, and the cerebral cortex, are not properly evaluated if the scan of the fetal skull is performed only in the axial planes^(^^5^^)^. Therefore, in 1996, Timor-Tritsch et al.^(^^6^^)^ described a fetal neurosonography technique that involves multiplanar analysis of the fetal brain structures, incorporating sagittal and coronal views of the fetal skull. The technique allows a detailed analysis of the cerebral anatomy and should be performed by properly trained professionals using high-resolution ultrasound to achieve diagnostic accuracy similar to that of fetal magnetic resonance imaging performed under ideal conditions^(^^5^^)^.

The International Society of Ultrasound in Obstetrics and Gynecology (ISUOG) has issued guidelines for the ultrasonographic study of the brain and spine in fetuses. The ISUOG guidelines are divided into two categories: basic CNS assessment; and neurosonographic evaluation^(^^4^^)^. The objective of this review was to describe, on the basis of the ISUOG guidelines, how the ultrasonographic evaluation of the fetal CNS should be performed.

## BASIC EVALUATION OF THE FETAL BRAIN

The basic evaluation of the fetal brain is that routinely used in the second-trimester fetal morphology scan, performed by the transabdominal approach. That technique is used in order to screen for CNS malformations in the second and third trimesters of gestation in low-risk patients, with a sensitivity of 80% for the detection of CNS malformations, and involves the study of structures of the fetal brain and spine^(^^4^^)^.

For an adequate basic ultrasonographic evaluation of the fetal brain morphology, it is necessary to perform scans in three axial planes of the skull-the transthalamic, transventricular, and transcerebellar planes. The structures evaluated in this step include the skull (for shape and biometry), the cerebral parenchyma (for texture), the lateral ventricles, the choroid plexus, the interhemispheric fissure, the cavum septum pellucidum (CSP), the thalami, the cerebellum, and the cisterna magna^(^^4^^)^.

**Figure 1:**
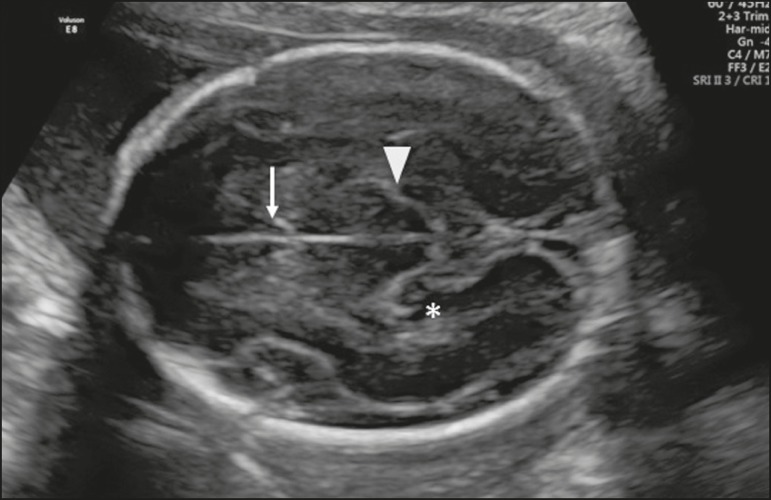
Axial view of the fetal head in the transthalamic plane, showing the CSP (arrow), the thalami (arrowhead), and the hippocampal gyrus (asterisk).

In the transthalamic plane, the biometry of the skull (biparietal diameter and cranial circumference) is performed using the following landmarks: the frontal horns of the lateral ventricles, the CSP, the thalami, and the hippocampal gyrus (Figure 1).

**Figure 2:**
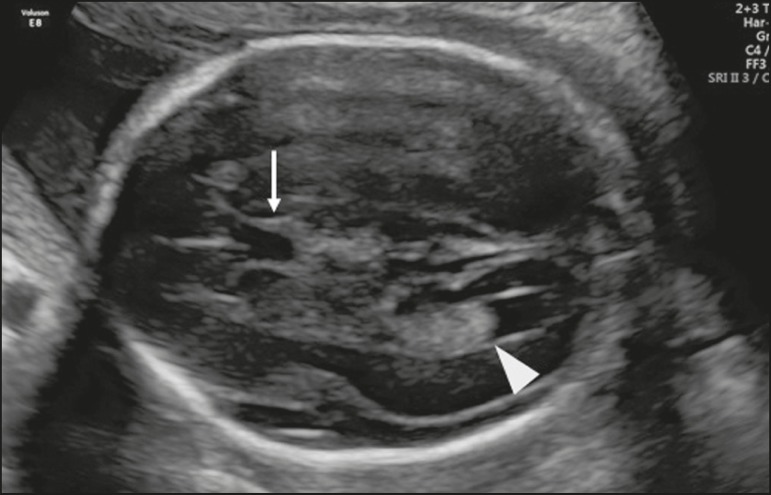
Axial view of the fetal head in the transventricular plane, showing the CSP (arrow) and the posterior horn of the lateral ventricle (arrowhead).

**Figure 3:**
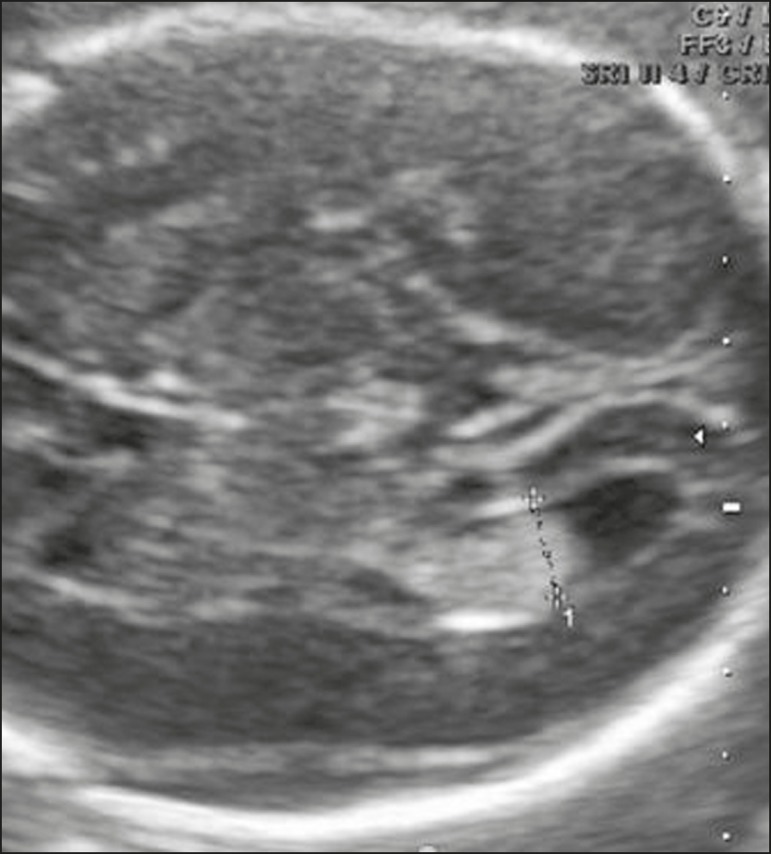
Measurement of the atrium of the lateral ventricle in the axial and transventricular planes, with the calipers positioned on the inner walls of the atrium, at the level of the glomus of choroid plexus.

In the transventricular plane, the posterior horn of the lateral ventricle (atrium) is measured using the following landmarks: CSP and posterior horns of the lateral ventricles-atrium (Figure 2). Some particularities of the structures can be analyzed in the transventricular plane. The CSP should be visualized on ultrasound between 17 and 37 weeks of gestation, and the absence of the CSP during that period may be a sign of an anomaly, including CSP agenesis, lobar holoprosencephaly, agenesis of the corpus callosum, severe hydrocephalus, and septo-optic dysplasia^(^^7^^)^. The measurement of the lateral ventricle atrium is recommended during gestation because its dilation (ventriculomegaly) is a common marker of CNS malformations. It should be measured at the level of the glomus of the choroid plexus, perpendicular to the ventricle cavity, and at the level of parieto-occipital fissure (which is identified from 20 weeks of gestation), with the calipers positioned on the inner portion of the walls of the lateral ventricles (Figure 3). That measure should remain stable throughout pregnancy and is considered normal at < 10 mm^(^^8^^)^.

**Figure 4:**
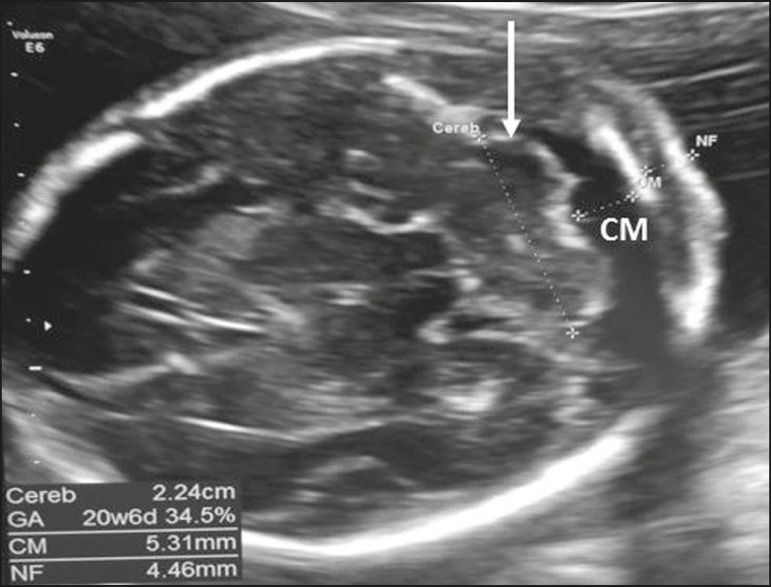
Axial view of the fetal head in the transcerebellar plane, showing the cerebellum (arrow) and the cisterna magna (CM).

In the transcerebellar plane, the posterior fossa structures (cerebellum and cisterna magna) are assessed using the following landmarks: the anterior horns of the lateral ventricles, the CSP, the thalamus, the cerebellum, and the cisterna magna. The cerebellum appears as a butterfly-shaped structure formed by two hemispheres that are connected by a central structure, which shows high echogenicity (comparable to that of the cerebellar vermis). As depicted in Figure 4, the transverse diameter of the cerebellum should be measured, as should the size of the cisterna magna, the latter being measured between the cerebellar vermis and the inner wall of the occipital bone, with a normal range of 2-10 mm^(^^9^^)^. It should be borne in mind that the cisterna magna may contain thin septations, which should not be confused with vascular structures or cystic malformations. Another point to be considered is that at early gestational ages (< 20 weeks of gestation), the cerebellar vermis may not completely cover the 4th ventricle, giving the false impression of a defect in the vermis. After week 20, that finding is suggestive of malformation of the posterior fossa^(^^10^^)^.

## NEUROSONOGRAPHIC EVALUATION

The complexity of CNS development and malformations, together with the limitations of fetal brain evaluation, which is routinely performed only in the axial planes (acoustic shadowing therefore being visualized only in the hemisphere proximal to the transducer, with inadequate evaluation of the midline structures and cerebral cortex), led to the emergence of the concept of fetal neurosonography. Consisting in a detailed ultrasonographic examination of the CNS, fetal neurosonography is based on the principle of multiplanar analysis of the brain structures, which is obtained by positioning the transducer in the cranial sutures and fontanelles^(^^4^^)^. For fetuses in cephalic (normal) presentation, a transabdominal or transvaginal approach can be used (the transvaginal approach offers the advantage of allowing the vaginal transducer to be used at a high frequency, which results in better image resolution). For fetuses in oblique presentation, the most common approach is transfundal, in which the transducer is positioned parallel to the abdomen. This has been shown to be an accurate method for the diagnosis of congenital CNS disorders. The method has been indicated for use in fetuses at risk for cerebral abnormalities (those with consanguineous parents, those with a family history of cerebral malformations or genetic diseases, and those with congenital infections, as well as those whose mothers used medications with teratogenic potential or were exposed to radiation or chemical substances after becoming pregnant) and for fetuses under diagnostic suspicion of CNS malformations after a routine prenatal ultrasound examination. Fetal neurosonography requires high-resolution ultrasound equipment and deep knowledge on the part of the professionals who perform it to study the embryology and anatomy of the CNS, in order to identify the manifestations of cerebral malformations and to gain knowledge of the physiopathology of CNS anomalies so that parents can be given appropriate counseling regarding the prenatal follow-up, treatment, and prognosis.

The neurosonographic examination includes the evaluation of the axial planes already described in the basic CNS assessment, adding to the evaluation in the coronal and sagittal planes. For the purpose of standardization, a systematic neurosonographic evaluation includes visualization in four coronal planes and three sagittal planes^(^^4^^)^.

### Coronal planes

**Figure 5:**
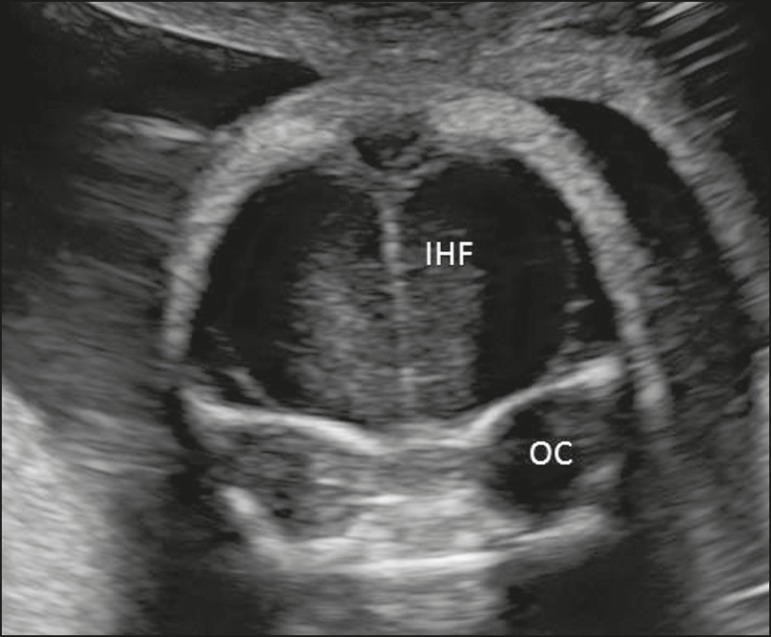
Coronal view of the fetal head in the transfrontal plane, showing the interhemispheric fissure (IHF), the frontal cortex, and the ocular orbits (OC).

**Figure 6:**
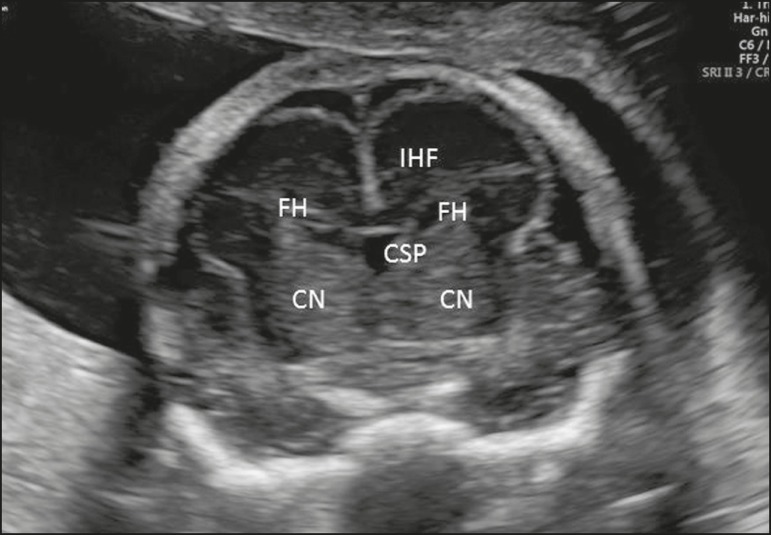
Coronal view of the fetal head in the transcaudate plane, showing the interhemispheric fissure (IHF), the CSP, the frontal horns of the lateral ventricles (FH), and the caudate nuclei (CN).

**Figure 7:**
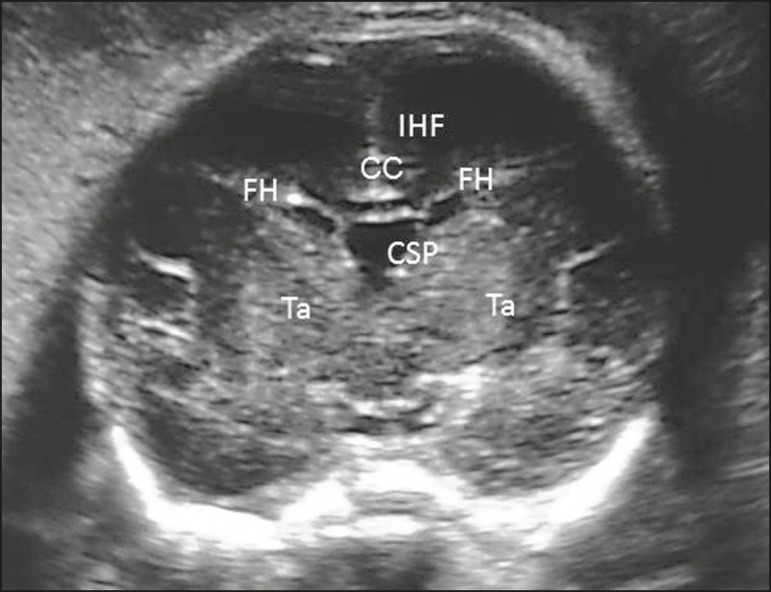
Coronal view of the fetal head in the transthalamic plane, showing the interhemispheric fissure (IHF), the CSP, the corpus callosum (CC), the frontal horns of the lateral ventricles (FH), and the thalami (Ta).

**Figure 8:**
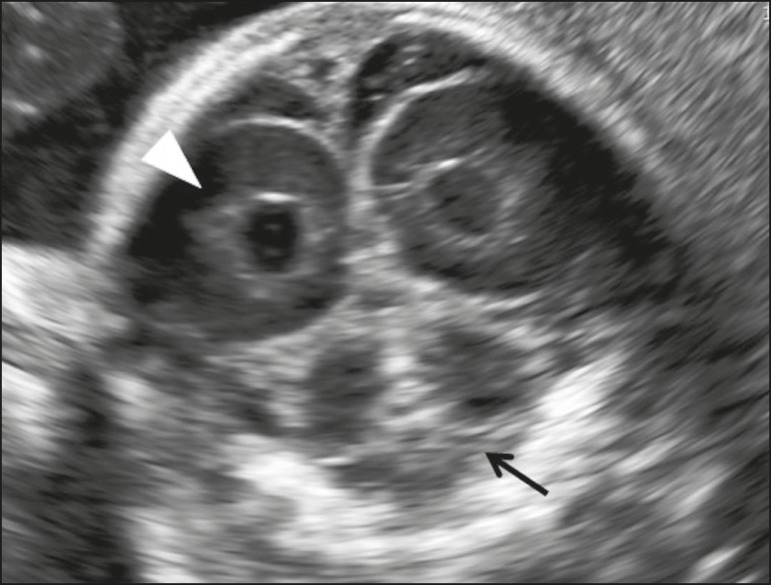
Coronal view of the fetal head in the transcerebellar plane, showing the posterior horns of the lateral ventricles (arrowhead) and the cerebellum (arrow). Note the differentiation between the cerebellar hemispheres and the vermis in the more echogenic central portion of the cerebellum.

The most relevant coronal planes are the transfrontal, transcaudate, transthalamic, and transcerebellar planes. In the transfrontal plane, visualized via the anterior fontanelle the following structures are analyzed: the interhemispheric fissure, the frontal cortex, the frontal horns of the lateral ventricles, the ocular orbits, and the sphenoid bone of the skull (Figure 5). In the transcaudate plane, the following are analyzed: the caudate nuclei, the interruption of the interhemispheric fissure by the anterior portion of the corpus callosum, the CSP, the frontal horns of the lateral ventricles, and the Sylvian fissure bilaterally (Figure 6). In the transthalamic plane, the following are analyzed: the thalami, the interruption of the interhemispheric fissure by the anterior portion of the corpus callosum, the CSP, the frontal horns of the lateral ventricles, the foramen of Monro, the third ventricle, the vessels forming the circle of Willis, and the optic chiasm near the base of the skull (Figure 7). In the transcerebellar plane, the posterior fontanelle is visualized for the assessment of the occipital horns of the lateral ventricles and the interhemispheric fissure. The hemispheres of the cerebellum and the cerebellar vermis are both identified in this plane, which is the ideal plane to differentiate the cerebellar hemispheres from the vermis, especially in cases of suspected vermis agenesis. In this plane, it is also possible to evaluate the calcarine fissure, which appears at approximately 22 weeks of gestation (Figure 8). The coronal planes are also useful for the evaluation of the subarachnoid space, the superior sagittal sinus, and the sagittal suture^(^^5^^)^.

### Sagittal planes

**Figure 9:**
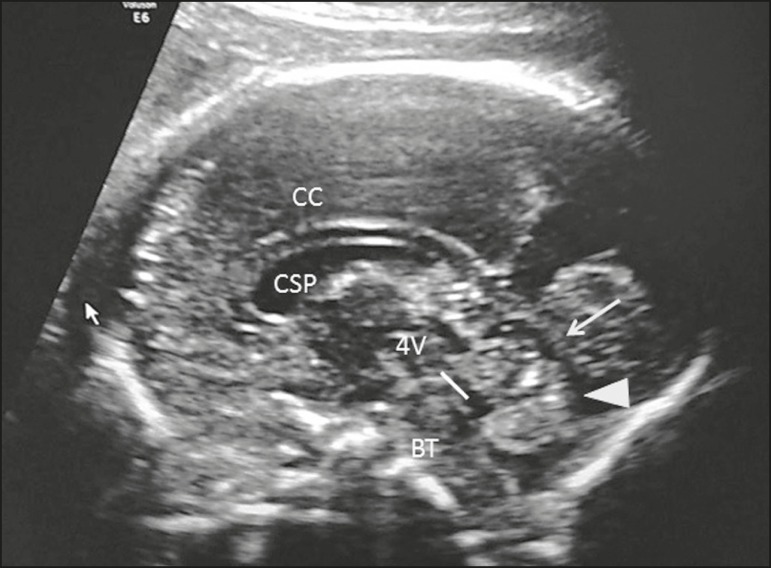
Sagittal view of the fetal head in the median sagittal plane, showing the corpus callosum (CC), the CSP, the cerebellar vermis (arrowhead), the tentorium (arrow), the fourth ventricle (4V), and the brain stem (BT).

**Figure 10:**
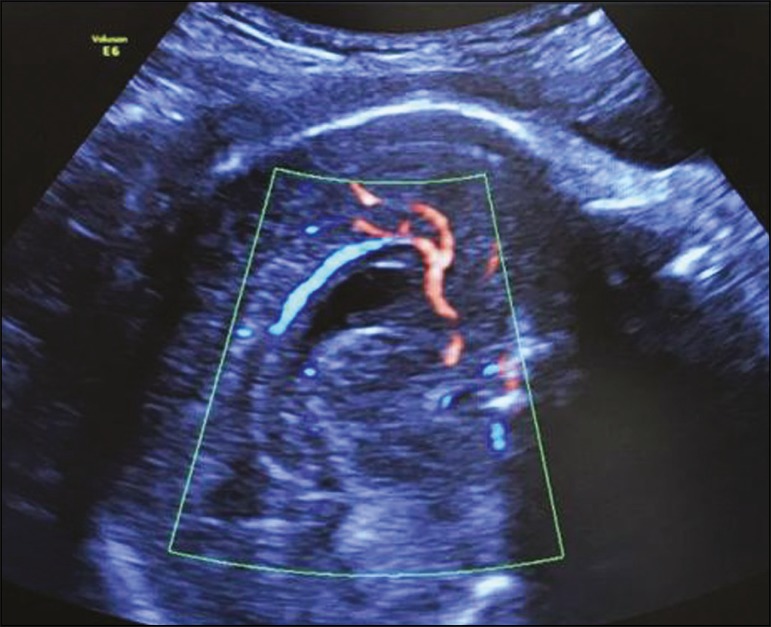
Sagittal view of the fetal head in the median sagittal plane, showing the pericallosal arteries and their branches.

**Figure 11:**
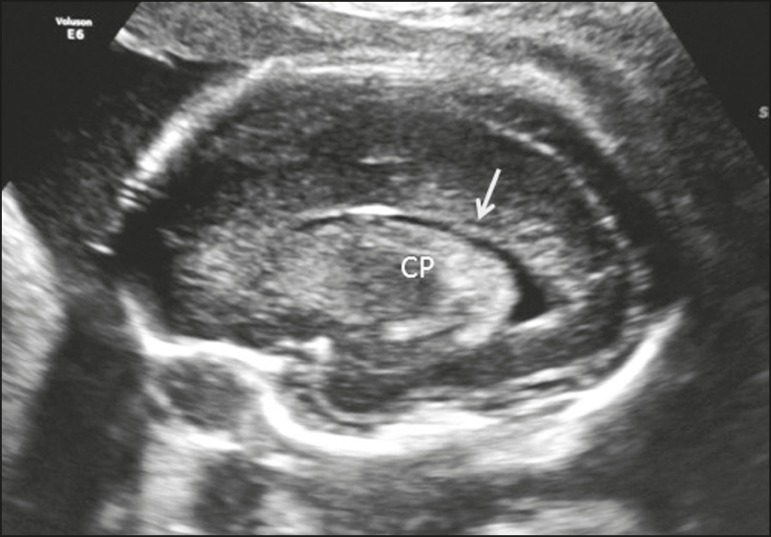
Sagittal view of the fetal head in the parasagittal plane, showing the three-horn view, the choroid plexus (CP), and the periventricular parenchyma (arrow).

Three sagittal planes are usually studied: the median sagittal plane; and the right and left parasagittal planes. The median sagittal plane is an important plane for the evaluation of the midline structures of the brain, including that of the corpus callosum (morphology and biometry), CSP, cavum vergae, cavum velum interpositum, brainstem, cerebellar vermis (morphology and biometry), cistern magna, and tentorium, as well as the cingulate fissure, which appears as an echogenic line above the corpus callosum at 24 weeks of gestation (Figure 9). Using color Doppler, the anterior cerebral artery, the pericallosal arteries (and their branches), and the vein of Galen can be visualized (Figure 10). The median sagittal plane is important for the differential diagnosis of midline and posterior fossa malformations. In the parasagittal plane, it is possible to visualize the entire lateral ventricle (three-horn view), the lateral ventricle wall, the periventricular parenchyma, and the cortex (Figure 11). In addition to the analyses mentioned above, the neurosonographic evaluation should include a detailed analysis of the cerebral cortex and the cerebral convolutions. The neurosonographic evaluation can be complemented by three-dimensional (3D) and color Doppler ultrasound. The 3D ultrasound allows a multiplanar study of the cerebral structures using various software-based approaches (multiplanar mode, tomography ultrasound imaging, volume contrast imaging, OmniView, virtual organ computer-aided analysis, and power Doppler 3D imaging), as well as offering the possibility of offline analysis through storage of 3D volumes. Color Doppler allows the study of the vascular anatomy and the blood flow in the brain, as well as the diagnosis of cerebral vascular malformations.

## EVALUATION OF THE FETAL SPINE

Evaluation of the spine is a part of the ultrasound examination of the fetal CNS. The fetal spine should be evaluated in the basic assessment and in the neurosonographic examination. It is recommended that full scans of the fetal spine be performed in the sagittal, coronal, and transverse plane, focusing on the vertebral bodies, the medullary canal, and the medulla itself. The integrity of the spine is inferred by the regular arrangement of the ossification centers of the vertebrae and the presence of the skin covering the full extent of the spine.

**Figure 12:**
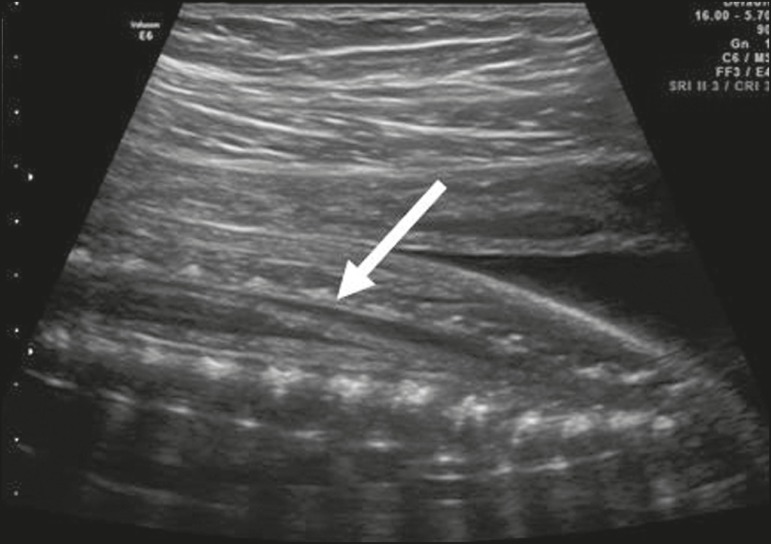
Sagittal view of the spine, showing the conus medullaris (arrow) located between the second and third vertebrae at 24 weeks of gestation.

The level of the conus medullaris may also be evaluated in the sagittal plane (Figure 12). The position of the conus medullaris changes during pregnancy. Recent studies have identified the fetal conus medullaris at the level of the fourth lumbar vertebra at 13-18 weeks of gestation, at the level of the third lumbar vertebra at 20-24 weeks, and at the level of the second lumbar vertebra at 40 weeks^(^^11^^)^.

## CONCLUSION

The systematization of fetal ultrasound examination of the CNS is fundamental for adequate professional training and diagnosis of its malformations. Fetal neurosonography is a diagnostic method that allows the detailed study of the fetal CNS anatomy and is indicated in situations of risk or suspicion of CNS malformations and should therefore be performed by properly trained professionals using high-resolution ultrasound equipment.
